# Inter-Pregnancy Weight Change and the Risk of Recurrent Pregnancy Complications

**DOI:** 10.1371/journal.pone.0154812

**Published:** 2016-05-04

**Authors:** Jacqueline M. Wallace, Sohinee Bhattacharya, Doris M. Campbell, Graham W. Horgan

**Affiliations:** 1 Lifelong Health Division, Rowett Institute of Nutrition and Health, University of Aberdeen, Aberdeen, Scotland, United Kingdom; 2 Dugald Baird Centre for Research on Women’s Health, Aberdeen Maternity Hospital, Aberdeen, Scotland, United Kingdom; 3 Biomathematics & Statistics Scotland, Rowett Institute of Nutrition and Health, University of Aberdeen, Aberdeen, Scotland, United Kingdom; Stellenbosch University, SOUTH AFRICA

## Abstract

Women with specific adverse pregnancy outcomes in their first pregnancy may be receptive to inter-pregnancy weight management guidance aimed at preventing these complications reoccurring in subsequent pregnancies. Thus the association between inter-pregnancy weight change and the risk of recurrent pregnancy complications at the second pregnancy was investigated in a retrospective cohort study of 24,520 women with their first-ever and second consecutive deliveries in Aberdeen using logistic regression. Compared with women who were weight stable, weight loss (>2BMI units) between pregnancies was associated with an increased risk of recurrent small for gestational age (SGA) birth and elective Cesarean-section, and was protective against recurrent pre-eclampsia, placental oversize and large for gestational age (LGA) birth. Conversely weight gain (>2BMI units) between pregnancies increased the risk of recurrent gestational hypertension, placental oversize and LGA birth and was protective against recurrent low placental weight and SGA birth. The relationships between weight gain, and placental and birth weight extremes were evident only in women with a healthy weight at first pregnancy (BMI<25units), while that between weight gain and the increased risk of recurrent gestational hypertension was largely independent of first pregnancy BMI. No relationship was detected between inter-pregnancy weight change and the risk of recurrent spontaneous preterm delivery, labour induction, instrumental delivery, emergency Cesarean-section or postpartum hemorrhage. Therefor inter-pregnancy weight change impacts the risk of recurrent hypertensive disorders, SGA and LGA birth and women with a prior history of these specific conditions may benefit from targeted nutritional advice to either lose or gain weight after their first pregnancy.

## Introduction

Serious pregnancy complications including hypertensive disorders, preterm birth, low birthweight and small for gestational age (SGA) birth occur most frequently in primiparous compared with multiparous women [1–3]. Nevertheless, women affected by these complications during their first pregnancy are at increased risk of these complications recurring subsequently. Typically in women with a history of pre-eclampsia the incidence of recurrent hypertensive disease varies from 11–65% [4–6], while the probability of a second spontaneous preterm birth is 3 to 4 fold- higher after an early first-delivery compared with women whose first deliveries were at term [7–9]. Likewise, women with a history of delivering a small neonate are reported to have a 4–8 fold greater risk of recurrent SGA or low birthweight in the next pregnancy compared with women whose firstborns grew normally *in utero* [10–13].

The pathophysiology underlying these recurrent and heterogeneous pregnancy complications likely involves intrinsic and extrinsic (environmental) influences. Of the potentially modifiable environmental factors there is evidence for a variable role of maternal smoking, inter-pregnancy interval, socioeconomic status and quality of antenatal care [14–17]. Maternal BMI has a profound impact on pregnancy outcome [18,19], and previous analyses show that inter-pregnancy weight change in both directions variously alters the incident or primary risk of experiencing a range of pregnancy complications at the second pregnancy including hypertensive disease, stillbirth, premature delivery, extremes of placental weight, SGA and large for gestational age (LGA) birth [3, 20–25]. However, there is a paucity of studies evaluating the relationship between inter-pregnancy weight change and recurrent pregnancy complications and this has been addressed herein in order to better inform weight management guidance in women with both a healthy and unhealthy weight at first maternity. Our analysis suggests that appropriate weight management between pregnancies has the potential to modify the recurrent risk of hypertensive disorders, SGA and LGA birth in women with a prior history of these specific conditions.

## Methods

### Study Population

This was a retrospective cohort study using data from the Aberdeen Maternity and Neonatal Databank (AMND). Data were entered by dedicated coding staff into a computerized database. Consistency checks and verification against case-notes ensured data validity. This involved checking completeness of data entry against NHS returns monthly and constant data cleaning and validation against case notes reported quarterly by the Data Management team to the AMND Steering Committee. Ethical approval was granted by the North of Scotland Research Ethics Service (REC Ref 13/NS/0050) for observational studies using routinely collected anonymized data from AMND, provided permission was granted by the Steering Committee (Caldicott guardians). After obtaining permission, data were extracted for all singleton births after 24 weeks’ gestation in Aberdeen city and district from 1986 and 2013. The population was women who had their first-ever and second consecutive births in Aberdeen, who booked for antenatal care before 24 weeks gestation on both occasions and whose height and weight were measured and recorded at the booking visit. After excluding women (n = 1501) with missing data for key variables (primarily placental weight), a final population of 24,520 was available for analysis.

### Study Design

Maternal weight at first antenatal visit for each pregnancy was adjusted to take into account stage of gestation when weight was measured, using maternal conformation data for women from the same geographical area [26]. Briefly this involved obtaining the z score of weight for height (difference between weight and mean weight for height, relative to standard deviation), adjusting this by adding a constant that depended on stage of gestation, and then recalculating a weight using only this adjusted z score. The resulting corrected weight was then used with the unadjusted height to calculate an adjusted BMI (weight/height^2^). Although the mean individual difference in gestational age at the initial hospital visit in the first versus the second pregnancy was small (1 ± 4 weeks, mean ± sd) this approach meant that maternal BMI calculated for both pregnancies was corrected to a standard stage of gestation for all women studied. Moreover as a small percentage of women had their first hospital visit after 16 weeks gestation (12 and 7% of women in first and second pregnancies), this approach facilitated inclusion of women who had a first booking appointment up to 24 weeks gestation. These adjusted maternal BMIs were used to calculate the inter-pregnancy change in BMI and women were categorized as follows: women who decreased their BMI by >2units (BMI/weight loss group), women who increased their BMI by >2units (BMI/weight gain group), and women who maintained their BMI within a 2unit loss or gain (BMI/weight stable = reference group). Initial BMI at the beginning of the first pregnancy was categorized using conventional cut-offs for underweight (<18.5), normal weight (18.5–24.9), overweight (25.0–29.9) or obese (≥30). The inter-delivery interval was calculated in years between the birth of the first and second child. Other covariates including maternal age, height and smoking habit were grouped as detailed in the relevant table.

The focus was pregnancy complications that recur relatively frequently at first and second pregnancy, thus rarely recurring complications such as stillbirth and neonatal mortality were not considered. The pregnancy complications and obstetric outcomes assessed included pre-eclampsia and gestational hypertension (coded according to ISSHP definition), postpartum hemorrhage (defined as blood loss of >500ml or 1000ml at vaginal or Cesarean delivery, respectively), type of labor (spontaneous or induced or elective Cesarean), type of delivery (spontaneous vaginal, instrumental, elective or emergency Cesarean) and spontaneous preterm delivery (<37weeks). Gestational age was recorded according to last menstrual period and was confirmed by ultrasound. Perinatal outcomes were birthweight and placental weight. The latter was weighed untrimmed and recorded to the nearest 10g. Birthweight was defined as SGA if weight was less than the 10^th^ centile or LGA if weight was above the 90^th^ centile for gestation using gender and parity specific birthweight charts for Scottish singleton births [27]. Similarly gestational age, gender and parity specific placental weight charts were used to define low (<10^th^centile) and high (>90^th^centile) placental weight [28].

### Statistical Analysis

The frequency of maternal characteristics at first maternity and complication incidence during first and second pregnancies only or in both pregnancies in relation to the BMI-change category between pregnancies was analyzed by Chi-Square (Table 1). Distribution of inter-pregnancy weight (BMI) change as a continuous variable was compared with maternal characteristics and pregnancy outcomes as categorical predictors using one-way ANOVA followed posthoc by Tukey’s method. The effect of initial BMI category at first pregnancy and BMI change category between pregnancies on BMI/weight-change was also analyzed using one-way ANOVA (Table 2). In Table 3 the recurrent and incident risk of each pregnancy complication was compared by Chi-Square tests. The significance of any trend in the recurrence rate of each complication (i.e. whether detected in both pregnancies) in relation to the BMI change since the first pregnancy (Table 4) was evaluated by Cochran-Armitage test. The risk of specific pregnancy complications recurring in the second pregnancy in relation to inter-pregnancy BMI change were assessed using logistic regression (Table 4). Risks are presented as Odds Ratios (OR) with 95% confidence intervals (CI) and were adjusted for BMI and year of delivery at first pregnancy, height, inter-delivery interval, along with maternal age and smoking status, baby gender and gestational age at first and second delivery. Variables were additionally adjusted for the co-occurrence of either pre-eclampsia or gestational hypertension. After testing for an interaction between BMI category at baseline pregnancy and BMI change category, we investigated whether BMI at beginning of first pregnancy modified any relationship between inter-pregnancy BMI change and repeat pregnancy complication risk at second pregnancy by repeating the logistic regression for women with BMI below or above 25 at first pregnancy (Table 5). Cochran-Armitage test was performed using R (Foundation Statistical Computing, Vienna, Austria) and all other analysis using Minitab (v16; Minitab Inc., State College, PA).

**Table 1:** Maternal characteristics and distribution of pregnancy complications in relation to inter-pregnancy BMI change category and mean change in BMI.

	Inter-pregnancy BMI change category					
	(number of women)					
Maternal characteristics at first pregnancy and complication incidence during first and second pregnancies	<-2*	-2 to<2*	>2*	P Value^¥^	Mean change in BMI (sd)	P Value^$^
	(n = 1191)	(n = 17059)	(n = 6270)			
Age, years (number of women)						
≤19 (2839)	4.9	52.0	43.1	<0.001	2.14 (3.19)^a^	<0.001
20–24 (6826)	5.2	65.0	29.8		1.24 (2.56)^b^	
25–29 (8841)	4.8	73.4	21.8		0.83 (2.07)^c^	
30–34 (4956)	4.4	77.8	17.8		0.63 (1.81)^d^	
≥35 (1058)	4.8	76.3	18.9		0.63 (2.04)^cd^	
Height, cm						
≤159 (7464)	5.0	68.4	26.6	0.024	1.12 (2.41) ^a^	0.003
160–164 (7471)	4.5	69.5	26.0		1.06 (2.39)^ab^	
165–169 (5920)	5.0	70.4	24.6		0.98 (2.31)^b^	
≥170 (3665)	4.9	71.0	24.1		0.98 (2.31)^ab^	
Adjusted BMI, kg/m^2^						
≤18.5 (372)	0.3	71.5	28.2	<0.001	1.54 (1.99)^a^	<0.001
18.6–24.9 (14334)	2.0	77.0	21.0		0.96 (1.95)^b^	
25–29.9 (6979)	7.3	63.1	29.6		1.12 (2.58)^c^	
≥30 (2835)	13.9	47.8	38.3		1.26 (3.49)^ac^	
Smoking habit						
Non-smoker (16200)	4.4	71.3	24.3	<0.001	0.99 (2.28)^a^	<0.001
Smoker- 1^st^ pregnancy only (1490)	4.3	58.2	37.5		1.70 (2.59)^b^	
Smoker- 2^nd^ pregnancy only (776)	8	63.8	28.2		1.05 (2.86)^a^	
Smoker- both pregnancies (4344)	6.5	66.7	26.8		1.03 (2.53)^a^	
Not recorded (1710)	4	72.4	23.6		1.01 (2.24)^a^	
Inter-delivery interval (years)						
≤1 (2663)	5.1	76.0	18.9	<0.001	0.60 (1.98)^a^	<0.001
2 (7647)	5.4	77.7	16.9		0.56 (1.98)^a^	
3 (6275)	4.6	72.8	22.6		0.89 (2.06)^b^	
>3 (7935)	4.5	57.0	38.5		1.78 (2.83)^c^	
Pre-eclampsia						
Neither pregnancy (22816)	4.8	70.2	25.0	<0.001	1.02 (2.34)^a^	<0.001
First pregnancy only (1215)	5.3	64.3	30.4		1.23 (2.58)^ab^	
Second pregnancy only (306)	6.2	51.3	42.5		1.87 (3.03)^c^	
Both pregnancies (183)	4.4	58.4	37.2		1.69 (2.64)^bc^	
Gestational hypertension						
Neither pregnancy (18681)	4.9	71.0	24.1	<0.001	0.97 (2.31)^a^	<0.001
First pregnancy only (3958)	4.9	67.0	28.1		1.16 (2.39)^b^	
Second pregnancy only (1042)	4.0	61.6	34.4		1.66 (2.90)^c^	
Both pregnancies (839)	4.5	59.2	36.3		1.47 (2.61)^c^	
Induced labour						
Neither pregnancy (14402)	4.6	72.1	23.3	<0.001	0.95(2.23)^a^	<0.001
First pregnancy only (5350)	5.5	66.8	27.7		1.11(2.50) ^b^	
Second pregnancy only (2672)	4.9	65.8	29.3		1.29(2.52)^bc^	
Both pregnancies (2096)	5.1	63.8	31.1		1.36 (2.65)^c^	
Elective Cesarean						
Neither pregnancy (21147)	4.7	70.1	25.1	<0.001	1.03 (2.32)^a^	0.033
First pregnancy only (485)	2.9	75.5	21.6		0.91 (2.03) ^ab^	
Second pregnancy only (2468)	5.8	63.9	30.3		1.24 (2.78)^b^	
Both pregnancies (420)	8.1	66.7	25.2		0.87 (2.46)^ab^	
Emergency Cesarean						
Neither pregnancy (19544)	4.6	70.6	24.8	<0.001	1.03 (2.33)^a^	<0.001
First pregnancy only (3055)	6.2	66.8	27.0		1.04 (2.49)^a^	
Second pregnancy only (967)	4.1	60.9	35.0		1.54 (2.67)^b^	
Both pregnancies (954)	7.0	66.3	26.7		0.98 (2.37)^a^	
Instrumental delivery						
Neither pregnancy (16554)	5.1	69.1	25.8	0.014	1.05(2.42)	0.127
First pregnancy only (6220)	4.3	70.9	24.8		1.00(2.25)	
Second pregnancy only (1083)	4.6	70.2	25.2		1.08 (2.25)	
Both pregnancies (663)	3.0	69.2	27.8		1.21(2.15)	
Spontaneous preterm						
Neither pregnancy (21353)	4.8	69.8	25.4	0.195	1.04 (2.36)	0.636
First pregnancy only (1451)	4.3	70.0	25.7		1.03 (2.29)	
Second pregnancy only (1267)	5.3	66.5	28.2		1.19 (2.52)	
Both pregnancies (449)	6.0	68.2	25.8		1.08 (2.55)	
Antepartum hemorrhage						
Neither pregnancy (20324)	4.8	70.2	25.0	<0.001	1.02 (2.34)	0.004
First pregnancy only (2112)	4.4	67.3	28.3		1.17 (2.39)	
Second pregnancy only (1670)	5.7	66.3	28.0		1.16 (2.56)	
Both pregnancies (414)	6.0	62.6	31.4		1.19 (2.53)	
Birth wt. <10^th^ C SGA						
Neither pregnancy (20656)	4.7	69.5	25.8	<0.001	1.07 (2.36)^a^	<0.001
First pregnancy only (1790)	4.3	68.8	26.9		1.09 (2.24)^a^	
Second pregnancy only (1368)	7.0	70.0	23.0		0.83 (2.60)^ab^	
Both pregnancies (706)	5.7	74.3	20.0		0.76 (2.37)^b^	
Birth wt. >90^th^ C LGA						
Neither pregnancy (20707)	4.9	70.7	24.4	<0.001	0.99 (2.32)^a^	<0.001
First pregnancy only (1417)	6.1	65.0	28.9		1.08 (2.57)^a^	
Second pregnancy only (1583)	3.7	63.3	33.0		1.53 (2.52)^b^	
Both pregnancies (813)	4.7	61.7	33.6		1.46 (2.61)^b^	
Placental wt. <10^th^ C						
Neither pregnancy (21126)	5.0	68.9	26.1	<0.001	1.07 (2.37)^a^	<0.001
First pregnancy only (1704)	3.4	71.9	24.7		1.02 (2.21)^ab^	
Second pregnancy only (1325)	4.8	74.4	20.8		0.81 (2.49)^b^	
Both pregnancies (365)	2.7	80.3	17.0		0.78 (2.08)^ab^	
Placental wt. >90^th^ C						
Neither pregnancy (19301)	4.7	71.1	24.2	<0.001	0.99 (2.31)^a^	<0.001
First pregnancy only (2166)	6.2	66.1	27.7		1.07 (2.44)^a^	
Second pregnancy only (2136)	4.6	62.5	32.9		1.40 (2.57)^b^	
Both pregnancies (917)	5.2	62.6	32.2		1.39 (2.73)^b^	

*Values are percentage of women.

^¥^P values obtained from Chi-squared test of counts.

^$^P values from one-way ANOVA followed post-hoc by Tukey method.

For the latter, where superscript letters within a parameter differ, P<0.01.

**Table 2:** Average weight (and BMI) change between first and second pregnancy in relation to BMI category at first pregnancy and BMI change category.

	BMI category at first pregnancy				
BMI change category	Underweight	Normal	Overweight	Obese	^¥^P value
Loss < -2	-8.08 ^ß^ (-2.93)^abc^	-7.07±2.14 (-2.67±0.73)^a^	-8.10±3.12 (-3.05±1.11)^b^	-11.13±6.10 (-4.19±2.25)^c^	P<0.001
Stable -2 to <2	1.71±2.08 (0.65±0.78)^a^	0.78±2.49 (0.29±0.93)^b^	0.48±2.78 (0.18±1.04)^c^	0.59±2.89 (0.22±1.08)^bc^	P<0.001
Gain >2	10.22±6.20 (3.83±2.23)^abc^	9.83±5.42 (3.74±2.06)^a^	10.90±5.78 (4.12±2.15)^b^	11.95±6.46 (4.51±2.42)^c^	P<0.001

Values are mean ± sd, with weight in kg and BMI as kg/m^2^. Number of women per group can be derived from Table 1.

^¥^P values from one-way

ANOVA followed post-hoc by Tukey method for both weight and BMI change. For both parameters where superscripts differ within a row, P<0.01.

^ß^n = 1, so no deviation calculated.

**Table 3:** Frequency of recurrent pregnancy complications in women with previous history compared with incident rate in second pregnancy in women with no previous history.

Complication	Recurrent complication rate (previous history)	Incident complication rate in second pregnancy (no previous history)	P value*
Pre-eclampsia	183 of 1398 (13.1%)	306 of 23122 (1.3%)	**<0.001**
Gestational hypertension	839 of 4797 (17.5%)	1042 of 19723 (5.3%)	**<0.001**
Induced labour	2096 of 7446 (28.1%)	2672 of 17074 (15.6%)	**<0.001**
Elective cesarean	420 of 905 (46.4%)	2468 of 23615 (10.4%)	**<0.001**
Emergency cesarean	954 of 4009 (23.8%)	967 of 20511 (4.7%)	**<0.001**
Instrumental delivery	663 of 6883 (9.6%)	1083 of 17637 (6.1%)	**<0.001**
Spontaneous preterm	449 of 1900 (23.6%)	1267 of 22620 (5.6%)	**<0.001**
Antepartum hemorrhage	414 of 2526 (16.4%)	1670 of 21994 (7.6%)	**<0.001**
SGA, <10^th^ C	706 of 2496 (28.3%)	1368 of 22024 (6.2%)	**<0.001**
LGA, >90^th^ C	813 of 2230 (36.4%)	1583 of 22290 (7.1%)	**<0.001**
Placental Wt. <10^th^C	365 of 2069 (17.6%)	1325 of 22451 (5.9%)	**<0.001**
Placental Wt. >90^th^C	917 of 3083 (29.7%)	2136 of 21437 (10.0%)	**<0.001**

* P values obtained from Chi-squared test of counts

**Table 4:** Frequency rate and adjusted odds ratios for recurrent adverse perinatal outcomes in first and second consecutive pregnancies in relation to BMI change from first pregnancy.

		BMI change (no. of women, % study population)			
Complication both pregnancies		Loss, <-2†	Stable, -2 to <2	Gain, >2	Trend^¥^, P value
(cases,%)		(1191, 4.9%)	(17059, 69.6%)	(6270, 25.5%)	
Pre-eclampsia	Rate (%)	0.67	0.63	1.08	**0.002**
(n = 183)	OR (95% CI)	0.43(0.20–0.95)*	1	1.13(0.80–1.59)	0.058
Gestational hypertension	Rate (%)	3.19	2.91	4.84	**<0.001**
(n = 839)	OR (95% CI)	0.80(0.56–1.14)	1	**1.72(1.47–2.02**)***	**<0.001**
Induced labour	Rate (%)	8.90	7.84	10.39	<**0.001**
(n = 2096)	OR (95% CI)	0.94(0.75–1.16)	1	1.12(1.01–1.25)*	0.061
Instrumental delivery	Rate (%)	1.67	2.69	2.93	**0.040**
(n = 663)	OR (95% CI)	0.73(0.46–1.15)	1	1.08(0.89–1.30)	0.252
Elective cesarean	Rate (%)	2.85	1.64	1.69	0.148
(n = 420)	OR (95% CI)	1.56(1.05–2.31)*	1	0.96(0.75–1.22)	0.061
Emergency cesarean	Rate (%)	5.62	3.70	4.07	0.534
(n = 954)	OR (95% CI)	1.11(0.84–1.46)	1	1.00(0.85–1.17)	0.739
Spontaneous preterm	Rate (%)	2.26	1.79	1.85	0.709
<37wks, (n = 449)	OR (95% CI)	1.30(0.76–2.21)	1	0.78(0.58–1.04)	0.101
Antepartum hemorrhage	Rate (%)	2.09	1.52	2.07	0.062
(n = 414)	OR (95% CI)	1.17(0.76–1.80)	1	1.09(0.87–1.38)	0.632
SGA, <10^th^ Centile	Rate (%)	3.36	3.08	2.24	**<0.001**
(n = 706)	OR (95% CI)	1.77(1.26–2.50)***	1	0.73(0.59–0.89)**	**<0.001**
LGA, >90^th^ Centile	Rate (%)	3.19	2.94	4.35	**<0.001**
(n = 812)	OR (95% CI)	0.64(0.45–0.92)*	1	1.31(1.11–1.54)***	**<0.001**
Placental Wt. <10^th^ C	Rate (%)	0.84	1.72	0.99	**0.015**
(n = 365)	OR (95% CI)	0.81(0.42–1.55)	1	0.61(0.45–0.82)***	**0.004**
Placental Wt. >90^th^ C	Rate (%)	4.03	3.36	4.70	**<0.001**
(n = 917)	OR (95% CI)	0.73(0.53–1.00)*	1	1.22(1.04–1.42)*	**0.002**

Odds ratios (OR) and 95% confidence limits (CI) from logistic regression *P<0.05, **P<0.01 ***P<0.001 relative to stable BMI reference group (OR = 1). Models adjusted for baseline BMI and year of delivery at first pregnancy, height and inter-delivery interval, and maternal age, smoking status, gestational age and baby gender at first and second pregnancy. Where appropriate, variables were additionally adjusted for the co-occurrence of pre-eclampsia or gestational hypertension at first and second pregnancy.

^†^ Refers to BMI loss between pregnancies of >2 units.

^¥^Significance of trend for complication frequency rate from Cochran-Armitage test and for OR’s from overall Chi square value.

SGA = small for gestational age, LGA = large for gestational age. Birth and placental weight categories from gender and parity specific centile charts [27, 28].

**Table 5:** Frequency rate and adjusted odds ratios for adverse perinatal outcomes occurring in both first and second consecutive pregnancies in relation to BMI change from first pregnancy for women with BMI (a) <25 and (b) >25 units in first pregnancy.

		BMI change Category			
(a) Complication in both pregnancies with healthy BMI at first pregnancy		Loss†	Stable	Gain	Trend^¥^, P value
(cases)		<-2	-2 to <2	>2	
Pre-eclampsia	Rate (%)	0	0.42	0.67	**0.034**
(n = 69)	OR (95% CI)	n/a	1	1.28(0.72–2.27)	0.705
Gestational hypertension	Rate (%)	1.04	2.21	3.88	**<0.001**
(n = 374)	OR (95% CI)	0.45(0.14–1.42)	1	2.19(1.73–2.79)***	**<0.001**
Induced labour	Rate (%)	5.90	6.85	8.57	**<0.001**
(n = 1059)	OR (95% CI)	0.79(0.47–1.31)	1	1.11(0.94–1.30)	0.269
Instrumental delivery	Rate (%)	1.74	2.74	3.40	**0.024**
(n = 421)	OR (95% CI)	0.69(0.28–1.70)	1	1.17(0.92–1.49)	0.297
SGA, <10^th^ Centile	Rate (%)	7.29	3.73	2.79	**<0.001**
(n = 530)	OR (95% CI)	2.52(1.57–4.04)***	1	0.61(0.47–0.79)***	**<0.001**
LGA, >90^th^ Centile	Rate (%)	1.74	1.94	2.95	**<0.001**
(n = 315)	OR (95% CI)	0.76(0.31–1.88)	1	1.64(1.26–2.14)***	**0.001**
Placental Wt. <10^th^ Centile	Rate (%)	1.74	2.16	0.99	**<0.001**
(n = 280)	OR (95% CI)	1.15(0.47–2.85)	1	0.41(0.28–0.62)***	**<0.001**
Placental Wt. >90^th^ Centile	Rate (%)	3.12	2.31	3.01	0.070
(n = 364)	OR (95% CI)	1.14(0.58–2.26)	1	1.30(1.01–1.68)*	0.124
(b) Complication in both pregnancies with unhealthy BMI at first pregnancy					
(cases)					
Pre-eclampsia	Rate (%)	0.89	1.02	1.48	**0.044**
(n = 114)	OR (95% CI)	0.51(0.23–1.15)	1	1.09(0.71–1.67)	0.188
Gestational hypertension	Rate (%)	3.87	4.29	5.8	**0.001**
(n = 465)	OR (95% CI)	0.81(0.56–1.18)	1	1.42(1.15–1.74)***	**0.001**
Induced labour	Rate (%)	9.85	9.78	12.2	**0.002**
(n = 1037)	OR (95% CI)	0.96(0.75–1.23)	1	1.13(0.98–1.32)	0.185
Instrumental delivery	Rate (%)	1.66	2.59	2.47	0.418
(n = 242)	OR (95% CI)	0.70(0.41–1.21)	1	0.95(0.71–1.27)	0.449
SGA, <10^th^ Centile	Rate (%)	2.10	1.79	1.71	0.495
(n = 176)	OR (95% CI)	1.40(0.84–2.32)	1	0.99(0.70–1.40)	0.401
LGA, >90^th^ Centile	Rate (%)	3.65	4.92	5.74	**0.009**
(n = 497)	OR (95% CI)	0.60(0.41–0.88)**	1	1.12(0.91–1.37)	**0.007**
Placental Wt. <10^th^ Centile	Rate (%)	0.55	0.85	0.98	0.238
(n = 85)	OR (95% CI)	0.65(0.25–1.65)	1	1.07(0.66–1.72)	0.589
Placental Wt. >90^th^ Centile	Rate (%)	4.31	5.43	6.37	**0.011**
(n = 553)	OR (95% CI)	0.65(0.46–0.92)*	1	1.13(0.93–1.37)	**0.010**

Odds ratios (OR) and 95% confidence limits (CI) from logistic regression *P<0.05, **P<0.01 ***P<0.001 relative to stable BMI reference group (OR = 1). Models adjusted for baseline BMI and year of delivery at first pregnancy, height and inter-delivery interval, and maternal age, smoking status, gestational age and baby gender at first and second pregnancy. Where appropriate, variables were additionally adjusted for the co-occurrence of pre-eclampsia or gestational hypertension at first and second pregnancy.

^†^ Refers to BMI loss between pregnancies of >2 units.

^¥^Significance of trend for complication frequency rate from Cochran-Armitage test and for OR’s from overall Chi square value.

SGA = small for gestational age, LGA = large for gestational age. Birth and placental weight categories from gender and parity specific centile charts [27, 28]. For the BMI loss, stable and gain categories the distribution of the study population was 2.0, 76.8 and 21.2% for women with first pregnancy BMI of <25 (a) and 9.2, 58.6 and 32.2% for women with first pregnancy BMI of >25 (b).

## Results

### Inter-pregnancy change in BMI and pregnancy complication incidence

Overall, BMI increased from 25.0 ± 4.3 (mean ± sd) at first pregnancy to 26.0 ± 5.0 at second (P<0.001). This comprised 1191 (4.8%) women who lost >2 BMI units, 17059 (69.6%) who maintained their inter-pregnancy BMI within 2 units, and 6270 (25.6%) who gained >2 BMI units. Table 1 details the frequency of maternal characteristics at first pregnancy and complication incidence during first and second pregnancies in relation to these BMI change categories. The number of women per BMI change category was influenced by age, height, first pregnancy BMI, smoking habit and inter-delivery interval and accordingly all were adjusted for in the regression analysis. Likewise, the incidence of all complications except spontaneous preterm delivery varied by BMI change category. The relationship between BMI at first pregnancy and the direction of BMI change is worthy of comment as proportionately more initially overweight and obese women demonstrated greater weight fluctuations between pregnancies than the normal BMI group (Table 1). Further the magnitude of both weight/BMI loss and gain was greater in obese>overweight>normal (Table 2).

Irrespective of BMI change category, on average, weight change between first and second pregnancy decreased with age and height and increased with longer inter-delivery interval (Table 1). Women who smoked only in first pregnancy had higher inter-pregnancy weight gain than non-smokers, women who smoked in both pregnancies and women who smoked only in second pregnancy (P<0.01). The change in BMI between pregnancies was lowest in women with SGA birth in both pregnancies while weight gain between pregnancies was equally high in women with LGA birth and placental weight >90^th^centile in both pregnancies and at second pregnancy only. Inter-pregnancy weight gain in women with pre-eclampsia and hypertension in both pregnancies was intermediate between women experiencing these complications in second pregnancy only and first pregnancy only/neither pregnancy groups. The number of women experiencing individual complications in both pregnancies was low compared with the incident frequency in first or second pregnancy only (P<0.001 by Chi-squared test, for all except emergency Cesarean-section). Nevertheless, the recurrent pregnancy complication rate for women experiencing a specific complication at first pregnancy was significantly higher than the incident frequency at second pregnancy in women with no history of that complication (Table 3). This was striking for the relative frequencies of recurrent pre-eclampsia, preterm delivery, birthweight extremes and Cesarean-section, which were at least 4-fold higher than in previously non-complicated pregnancies.

In Table 4 the incidence rate and adjusted odds ratios for adverse perinatal outcomes at second pregnancy in relation to BMI change category between pregnancies, are presented for women experiencing the individual complication in the first pregnancy. Inter-pregnancy weight gain equivalent to >2 BMI units (mean ± sd, 4.0 ± 2.18) was associated with increased risk of recurrent gestational hypertension (OR 1.72, P<0.001), placental weight >90^th^ centile (OR 1.22, P<0.05) and LGA (OR 1.31, P<0.001), and was protective against recurrent risk of placental weight <10^th^ centile (OR 0.61, P<0.001) and SGA (OR 0.73, P<0.01). A modestly higher risk of induced labor was also evident (OR 1.12, P< 0.05). In contrast women who lost >2 BMI units (mean ± sd, 3.3 ± 1.64) between pregnancies were protected from pre-eclampsia recurring (OR 0.43, P<0.05) but had higher risk of experiencing a second elective Cesarean-section (OR 1.56, P<0.05) and SGA birth (OR 1.77, P<0.001). Weight loss was also protective against the recurrent risk of placental weight >90^th^ centile (OR 0.73, P<0.05) and LGA (OR 0.64, P<0.05). Weight gain or loss of >2 BMI units between pregnancies did not influence the recurrent risk of instrumental delivery, emergency Cesarean-section, spontaneous preterm delivery or antepartum hemorrhage. A small number of women with gestation hypertension at first pregnancy had pre-eclampsia in their second (171 of 3958, or 4.3%). For the population as a whole and relative to weight stable women, inter-pregnancy weight gain increased the risk of this scenario (OR 2.18 [95% CI 1.56–3.04], P<0.001).

### Pregnancy complications, baseline BMI category and inter-pregnancy weight change

The putative interaction between baseline BMI category and inter-pregnancy change in BMI category was examined and significant interactions (weight change effects differing between four baseline BMI categories) were evident for gestational hypertension (P = 0.01), LGA (P = 0.061) and placental weight <10^th^ centile (P = 0.023). On this basis and cognizant that this approach may miss detecting interactions for less prevalent outcomes, we sub-divided the data and compared effects of weight change between pregnancies on second pregnancy recurrent complication risk for women with BMI <25 (mean ± sd, 22.3 ± 1.71) versus BMI >25 (mean ± sd, 29.0 ± 3.97) at first pregnancy (Table 5). Data are shown for adverse outcomes where weight change was associated with significant linear trends across the BMI change spectrum and/or significant adjusted OR in at least one baseline BMI category only and thus spontaneous preterm delivery, elective or emergency Cesarean-section and antepartum hemorrhage are not included. Inter-pregnancy weight gain significantly increased the risk of gestational hypertension recurring in second pregnancy irrespective of maternal BMI at the first pregnancy and the risk was highest in women with healthy compared with unhealthy BMI at baseline (OR 2.19 vs.1.42, respectively). For women with healthy BMI at baseline, gaining >2 BMI units (mean ± sd, 3.7±2.06) between pregnancies also increased the risk of placental weight >90^th^ centile (OR 1.30, P<0.05) and LGA birth (OR 1.64, P<0.001) and was protective against placental weight <10^th^ centile (OR 0.41, P<0.001) and of experiencing a second SGA birth (OR 0.61, P<0.001). Conversely, women with a healthy BMI at baseline and an inter-pregnancy weight loss of >2 BMI units (mean ± sd, 2.7±0.73) had markedly higher risk of recurrent SGA (OR 2.52, P<0.001). None of these relationships were evident in women who were overweight at baseline. In the latter sub-analysis women who were overweight at baseline and lost >2 BMI units (mean ± sd, 3.5 ± 1.79) were protected against recurrent placental weight >90^th^ centile (OR 0.65, P<0.05) and LGA birth (OR 0.60, P<0.01). In both sub-cohorts the incidence rate of recurrent pre-eclampsia showed a linear trend across the BMI change spectrum (P = 0.034 and P = 0.044 in women who had healthy and unhealthy BMI at baseline) but the low incidence rate precludes meaningful risk assessment. The incidence rate of women with gestation hypertension in their first pregnancy and pre-eclampsia in their second was similarly low in both sub-cohorts (0.46 and 1.05% for healthy and unhealthy BMI groups). However for women with an unhealthy BMI at baseline, weight gain of >2 BMI units increased the risk of this scenario relative to those who remained weight stable (OR 2.40 [95% CI 1.56–3.68], P<0.001). Further sub-dividing these women into those who were overweight versus obese at baseline revealed that the increased risk of pre-eclampsia at the second pregnancy after gestational hypertension in the first was confined to initially overweight women who gained >2 BMI units in the inter-pregnancy period (OR3.04 [1.74–5.32], P<0.001) but failure to detect a similar relationship in obese women may be due to reduced power.

## Discussion

In this unselected population, women experiencing common pregnancy complications in their first-ever pregnancy were at increased risk of them recurring in their second pregnancy. This confirms findings for women with a history of hypertensive disorders, preterm delivery and SGA birth [4–13] and extends the scope to include LGA birth, placental weight extremes and type of delivery. The main objective was to establish if inter-pregnancy weight change in either direction plays a role in modifying the recurrent risk of any of these adverse pregnancy outcomes.

### Inter-pregnancy weight loss and recurrent pregnancy complications

Herein weight loss between pregnancies lowered recurrent pre-eclampsia risk by ~55%, as seen in a selected population of women with a history of the condition [29]. The effect of inter-pregnancy weight loss on pre-eclampsia risk generally is unclear. While weight loss of more than one BMI unit decreased primary risk of pre-eclampsia in second pregnancies in one large population by a modest 18% [20], it was without effect when normotensive obese or overweight women lost sufficient weight to descend a BMI category in another equally large study [22]. Taken together this suggests that the underlying biology differs. Women with a history of the condition may have persistent systemic inflammation which weight loss between pregnancies ultimately improves. In this recurrent risk analysis we elected to define BMI loss as >2 units rather than the one BMI unit change used previously to assess primary risk [3, 20]. While the former approach reduces the number of women in the BMI loss category it more rigorously defines what is likely to be real weight change. Accordingly if we assume an average height of 1.62m, a one BMI unit change corresponds to only 2.6kg which may be within the range of natural fluctuation whereas 2 BMI units is equivalent to just over 5kg. Indeed the average weight loss measured here was 8.5kg and is in theory an achievable target for women wishing to attenuate their recurrent risk of pre-eclampsia.

In direct contrast inter-pregnancy weight loss increased recurrent SGA risk by 77% in the study population as a whole. No comparable recurrent analysis has been reported but we previously observed an equivalent increase in primary SGA risk at second pregnancy in women with a healthy and unhealthy BMI at baseline pregnancy [3]. Herein the relationship between weight loss and recurrent SGA risk was significant only in women with a healthy weight at baseline (OR 2.52). Similarly in an independent population, BMI loss (>1 unit) in women with a healthy baseline BMI decreased fetal oversize but at the expense of a doubling of the risk of delivering a low birthweight (<2500g) infant [25], while in a population of obese women, 9% with previous SGA, only weight loss >8 BMI units increased SGA risk at second pregnancy irrespective of history [30]. Together these studies suggest that women with a history of SGA and a healthy BMI at the baseline pregnancy should avoid weight loss in the inter-pregnancy period.

The recurrent risk of spontaneous preterm delivery was independent of inter-pregnancy weight change. In contrast in an unselected population (n = 1241), women with previous preterm birth and who lost >5 BMI units between pregnancies had a higher crude incidence at the second pregnancy than women who gained weight or remained weight stable [9]. However, these authors did not differentiate between spontaneous versus medically-indicated preterm delivery or adjust for known confounders. Inter-pregnancy weight loss has been reported to increase the incident risk of spontaneous preterm delivery at the second pregnancy, while inter-pregnancy weight gain is modestly protective [3, 24]. Nevertheless our analysis suggests that weight change between pregnancies is unlikely to have a major influence on recurrent spontaneous preterm birth and intrinsic factors like cervical insufficiency and ethnicity are probably more important [31].

### Inter-pregnancy weight gain and recurrent pregnancy complications

Inter-pregnancy weight gain robustly increased the risk of recurrent gestational hypertension irrespective of baseline BMI. No recurrent risk has previously been reported but the adjusted odds ratio of 1.72 overall was equivalent to that reported between inter-pregnancy weight gain and the primary risk of hypertension at the second pregnancy in women from the same geographical area and in a much larger population wide study ([3, 20], OR 1.82 and 1.76, respectively). The striking similarity in these odds ratios implies that it is weight gain and the attainment of a higher BMI category at the start of the next pregnancy, rather than a women’s prior history that predicts hypertension risk; in support it is well established that the risk of pregnancy hypertensive disorders increases stepwise through normal to morbidly obese BMI categories in primiparous and multiparous women [19, 32, 33]. Indeed, although not strictly the focus of the current analysis, we also observed that weight gain increased the risk of women with gestational hypertension in their first pregnancy developing pre-eclampsia in their second. An analogous scenario probably underlies the observed association between weight gain and increased risk of recurrent LGA as birthweight likewise increases with greater BMI [19, 32, 33]. Herein weight gain protected against recurrent SGA birth, predominantly in women with BMI <25 at the baseline pregnancy. This relationship was paralleled by a protective effect of weight gain on the risk of recurrent low placental weight. Overall the implication is that normal or underweight women with a history of SGA may benefit from modest weight gain prior to conceiving their second child and the attenuated risk of a second SGA birth may partly be mediated by increased placental size. Placental weight increases 4.4g per BMI unit [33] and thus we propose that the increase in BMI between pregnancies has a positive effect on maternal nutritional status and nutrient reserves at the start of the second pregnancy, enhancing the placental growth trajectory and thereby fetal nutrient supply and ultimately growth. In partial support nutritional manipulation of weight and adiposity prior to conception (sheep paradigm) to achieve two contrasting maternal BMI categories has a profound influence on placental weight irrespective of gestational intake and is closely associated with birth weight at delivery [34].

### Strengths and weaknesses

Strengths included weight and height measurements at the first clinic appointment at a single maternity hospital on both occasions with weight adjusted to a standard gestational age for all maternities. This closely approximated pre-pregnancy BMI as it was recorded by trained staff thereby negating the recall bias associated with self-reported anthropometry. Further the analysis was adjusted for several known confounders. However maternal weight in late pregnancy was not routinely measured precluding differentiation of the impact of weight change during pregnancy versus after delivery and prior to the second conception. This is an unfortunate limitation of current UK healthcare practice. Further the population was relatively small with low ethnic diversity. The low recurrence rate for some complications may have limited power to detect all potential effects, particularly in the sub-analysis of women with a healthy versus unhealthy baseline BMI. Nevertheless the recurrence event rate for key complications (pre-eclampsia, premature delivery and SGA), agree with previous publications [4–13] by other groups using different populations. Although the data was collected over a time range where changes in obstetrical practice might have been expected (eg. criteria for cesarean section) this potential bias should have been minimized by including year of delivery in the adjusted model. Finally it is acknowledged that selected populations with specific maternity histories, namely hypertensive disorders, SGA or LGA birth, and larger numbers than available here are required to confirm the reported relationships with weight change.

## Conclusion

In conclusion, in an unselected population inter-pregnancy weight change impacts recurrent risk of hypertensive disorders, SGA and LGA birth and women with a prior history of these conditions may benefit from targeted nutritional guidance to either lose or gain weight after their first pregnancy. Women with a healthy BMI at the baseline pregnancy are on balance more sensitive to both potential risks and benefits associated with weight change; advice for preventing an individual recurrent complication should be cognizant of the potential impact of weight change on the incident risk of other pregnancy complications.
